# Advances in Polyhydroxyalkanoate (PHA) Production, Volume 3

**DOI:** 10.3390/bioengineering9070328

**Published:** 2022-07-19

**Authors:** Martin Koller

**Affiliations:** 1 Institute of Chemistry, University of Graz, NAWI Graz, Heinrichstrasse 28/IV, 8010 Graz, Austria; martin.koller@uni-graz.at; Tel.: +43-316-380-5463; 2 ARENA—Arbeitsgemeinschaft für Ressourcenschonende und Nachhaltige Technologien, Inffeldgasse 21b, 8010 Graz, Austria

**Keywords:** autotrophs, biopolyesters, CO_2_, cyanobacteria, industrialization, *mcl*-PHA, polyhydroxyalkanoate (PHA), polymer processing, polymer recovery, process design

## Abstract

Steadily increasing R&D activities in the field of microbial polyhydroxyalkanoate (PHA) biopolyesters are committed to growing global threats from climate change, aggravating plastic pollution, and the shortage of fossil resources. These prevailing issues paved the way to launch the third Special Issue of *Bioengineering* dedicated to future-oriented biomaterials, characterized by their versatile plastic-like properties. Fifteen individual contributions to the Special Issue, written by renowned groups of researchers from all over the world, perfectly mirror the current research directions in the PHA sector: inexpensive feedstock like carbon-rich waste from agriculture, mitigation of CO_2_ for PHA biosynthesis by cyanobacteria or wild type and engineered “knallgas” bacteria, powerful extremophilic PHA production strains, novel tools for rapid in situ determination of PHA in photobioreactors, modelling of the dynamics of PHA production by mixed microbial cultures from inexpensive raw materials, enhanced bioreactor design for high-throughput PHA production by sophisticated cell retention systems, sustainable and efficient PHA recovery from biomass assisted by supercritical water, enhanced processing of PHA by application of novel antioxidant additives, and the development of compatible biopolymer blends. Moreover, elastomeric medium chain length PHA (*mcl*-PHA) are covered in-depth, inter alia, by introduction of a novel class of bioactive *mcl*-PHA-based networks, in addition to the first presentation of the new rubber-like polythioester poly(3-mercapto-2-methylpropionate). Finally, the present Special Issue is concluded by a critical essay on past, ongoing, and announced global endeavors for PHA commercialization.

## 1. Introduction

Waste consisting of contemporarily used full carbon–carbon backbone plastics of fossil origin is very resistant to biodegradation, which generates enduring impairment to the marine and terrestrial environment [1]. Estimates of global emissions of plastic waste to fresh and sea water range from 9 to 23 million metric tons annually, the same order of magnitude as calculated for plastic waste emitted into the land. By 2025, these quantities are expected to double. All conventional plastic ever made either has been incinerated to energy and surplus CO_2_, or is still present on our planet; today, roughly 6 Gt of persistent macro-, micro-, and nano-plastic waste are accumulated in our ecosystems, also in living organisms and the trophic chains. These figures impressively illustrate that biobased and biodegradable solutions are indispensably needed [2].

As a potential remedy, so called “bioplastics” are strongly emerging today; however, we should handle this expression with care: Not all “bioplastics” are definitely “green” materials, hence, at the same time biobased, biosynthesized, biodegradable, and biocompatible. Moreover, “plastics”, by definition, are xenobiotic materials not occurring in nature, so the expression “bioplastics” is rather ambiguous. Poly(butylene adipate terephthalate) (PBAT), poly(lactic acid) (PLA), and thermoplastic starch (TPS) are the current “bioplastic” market leaders; for microbial polyhydroxyalkanoate (PHA) biopolyesters, a current share of not even 2% of the entire bioplastic market, reaching an annual production of not more than approximately 40,000 to 50,000 tons in 2021, is estimated. However, until 2026, the entire “bioplastic” production is expected to increase to about 7.6 million tons, more than twice the value for 2020. In 2026, PHA, the only truly biological (biobased and biosynthesized) and circular (biodegradable and compostable) group of materials among all products commercialized as “bioplastic” will already be among the top 5 “bioplastics”, with an expected share of 6.4% on the “bioplastics” market, which will correspond to about 0.5 million annual tons; hence, the tenfold production quantity of today is expected to be reached in just five years [3]!

With regard to these auspicious prospects, PHA can already be considered typical materials of “Industrial-” or “White Biotechnology”—hence, the large-scale manufacturing of bulk products starting from renewable resources by the action of living organisms (predominantly microbes) or parts thereof (enzymes). Large-scale PHA manufacturing will offer biodegradable materials for diverse packaging purposes in the food and non-food sector, as well as for numerous additional applications, such as in the pharmaceutical and biomedical field, for replacement of petrochemical microplastic in cosmetics, or even for bioremediation [4].

This positive development towards a relevant presence of PHA-based materials on the market in the near future is enabled by the enormous global efforts witnessed today in research and development on these materials. Concerted and synergistic actions of (system) biologists, microbiologists, (computational) biotechnologists, multi-omics experts, chemical engineers, material scientists, and market experts contribute to the rapid development of these microbial materials. The present third Special Issue for *Bioengineering* on advances in PHA production collects exemplary topical studies covering these fields; they give an excellent insight into the global progress in the field of these truly circular biopolyesters.

## 2. Individual Contributions

Since decades, the exploration of inexpensive feedstock for PHA production is regarded key to decrease the still too high PHA production price [5]. While considerable progress has already been achieved in this field, optimization of PHA production from inexpensive, abundantly available heterotrophic raw materials, especially of agro-industrial origin, from the greenhouse gas methane, and from surplus CO_2_, especially from industrial effluent gases, is still ongoing [6].

In the current Special Issue, organic second-generation raw materials from dairy industry, viticulture, and sugar beet processing were studied. Kiselev and colleagues investigated PHA biosynthesis by the wild type bacterial strain *Cupriavidus necator* B-10646 on enzymatic hydrolysates of sugar beet (*Beta vulgaris*) molasses; it was shown that the strain readily converted generated monomeric sugars for biomass growth and PHA biosynthesis. By establishing an optimized feeding strategy and culture medium composition, the authors were able to obtain up to 80 wt.% PHA in biomass. Interestingly, due to minor amounts of components present in hydrolyzed molasses acting as 3-hydroxyvalerate (3HV) precursors, the obtained PHA contained certain amounts of 3HV monomers, in addition to 3-hydroxybutyrate (3HB) as a main building block; composition of such high-quality PHA copolyesters with enhanced processability was finally fine-tuned by co-feeding appropriate amounts of precursor compounds [7].

Chang and colleagues utilized a surplus product from cheese and dairy industry for PHA production: Lactose present in raw cheese whey at a concentration of about 4–5 wt.% was biotechnologically converted in a first step to acetic acid by the aerobic bacterial strain *Acetobacter pasteurianus* C1. In a second step, generated acetic acid was transformed to the PHA homopolyester poly(3-hydroxybutyrate) (P(3HB)) by *Bacillus* sp. CYR-1, a novel Gram-positive PHA production strain. After the removal of excess protein from raw cheese whey, PHA production was increased to more than 400 mg/L. In their conclusion, authors emphasized that this two-stage process might be beneficial for conversion of such highly diluted feed streams like whey to PHA, without the need for whey pre-treatment by time-, energy-, and material-demanding lactose hydrolysis, and without prior ultrafiltration to concentrate lactose [8].

In the context of novel PHA production strains, such organisms thriving at extreme environmental temperature, salinity, heavy metal load, or pH-value conditions attract increasing attention for PHA biosynthesis under low energy requirements: They can be cultivated under such extreme conditions under restricted sterility precautions without risking the loss of cultivation batches due to microbial contamination [9]. Such concepts are currently termed “Next Generation Industrial Biotechnology” (NGIB) [10], and are already in use for PHA manufacturing by extremophilic engineered strains, mainly halophilic *Halomonas* sp., in inexpensive non-sterile media and simple, open, continuous cultivation batches [11]. In this aspect, Kourilova and co-workers converted aqueous extracts of grape pomace, an agro-industrial waste product form viticulture, to P(3HB); the thermophilic strain *Tepidimonas taiwanensis* LMG 22826 was used as production strain for these cultivation experiments. PHA production capacity of this organism was studied both on the level of phenotype and genotype. It turned out that the strain harbors a Class I PHA synthase similar to other well-known short-chain-length PHA (*scl*-PHA) producers like *C. necator*. Best results for P(3HB) production by this strain were obtained using extract of pomace remaining from pressing Veltliner grapes; here, almost 5 g/L biomass, containing about 48 wt.% P(3HB), were achieved within 72 h of cultivation at a thermophilic cultivation temperature of 50 °C, which is far above the typically mesophilic temperature optimum of established PHA production strains and most potential microbial contaminants [12].

In the context of autotrophic PHA biosynthesis, Lambauer and Kratzer studied P(3HB) homopolyester formation by the well-known aerobic non-phototrophic hydrogen-oxidizing strain *C. necator* H16 on mixtures of the gases H_2_, O_2_, and CO_2_. Special emphasis in this study was dedicated to safety aspects when handling such explosive “knallgas” mixtures according to valid safety standards and Good Laboratory Practice principles; this was realized by operating the process in a simple explosion-proof lab-scale bioreactor placed in a stable and secure way in a fume hood. Cultivations were carried out by supplying the carbon source CO_2_ and the reducing agent H_2_ in excess, while controlling O_2_ availability as the growth-determining factor. Remarkably, best results for P(3HB) biosynthesis were obtained when keeping the gas composition within the explosion limits of O_2_/H_2_ mixtures. About 13 g/L biomass, containing an expedient P(3HB) mass fraction of 80% was generated during 90 h of cultivation. Notably, no temperature- or pH-control was needed in these cultivation setups. This promising outcome might make this study pioneering for further autotrophic P(3HB) production batches by non-phototrophs, also on larger scale and by using industrial effluent gas [13]. A similar study by Tanaka *et al.* investigated the autotrophic biosynthesis of the readily marine biodegradable PHA copolyester poly(3-hydroxybutyrate-*co*-3-hydroxyhexanoate) (P(3HB-*co*-3HHx)), a PHA copolyester of increasing industrial significance to produce various single-use plastic articles, by differently engineered *C. necator* strains. Also in this study, CO_2_ was used as the sole carbon source. These cultivations were carried out in simple Erlenmeyer flaks, equipped merely with a sterile filter, a tube, and a pinchcock. P(3HB-*co*-3HHx) was accumulated at high intracellular fractions, with high molar shares of 3HHx found in obtained copolyester samples. Finally, it was possible to control the 3HHx fraction in P(3HB-*co*-3HHx) to a value of 11 mol%, which is desired for optimum processability of the material; this was achieved by transforming cells with the plasmid pBPP-ccr_Me_J_Ac_-emd harboring *A. caviae* *scl*-PHA specific PhaJ [14]. In addition to non-photoautotrophic “knallgasbacteria” able to build up new biomass from CO_2_ and H_2_, the oxygenic group of cyanobacteria is able to fix CO_2_ phototrophically by resorting to the energy provided by sunlight (“photooxybacteria”). Beside other marketable bioproducts like precious pigments (especially phycobilins) or diverse bioactive compounds, many cyanobacteria are also described as proficient photoautotrophic PHA producers [15]. Therefore, the quest for new, robust PHA-accumulating cyanobacteria, adapted to the most diverse habitats, is steadily ongoing. In a long-term study, Meixner and associated researchers took samples from 25 different locations as remote as a geyser in Iceland, an Argentinian glacier lake, a snow field in Greenland, a wet rock in the Dolomite mountains, and a dammed lake at the Iberian Peninsula. A total of five years was dedicated by the authors to sample collection, strain isolation and purification by sophisticated methods like antibiotics supply and benefitting from cyanobacteria´s phototaxis, identification, and characterization of new microbial strains. These isolation studies resulted in a total of 71 primary cultures, 34 of which, containing 19 different cyanobacteria, were thriving in a stable manner after multiple transfers into a fresh medium. Four among these isolated cyanobacteria were shown to accumulate PHA in a simple home-made tubular photobioreactor, and two among them were successfully cultivated even under non-axenic, open cultivation conditions without the need for artificial light supply. Surprisingly, it was strain *Synechocystis* sp. IFA-3, an organism isolated from a fire pond just around the corner of the researchers’ institute near Vienna, which turned out to hold highest promise as proficient new PHA producer among all organism isolated by the authors from the magnitude of samples collected all over the world [16]. Also in the nexus of cyanoabacteria, Doppler et al. developed a new in situ method for fast and reliable determination of PHA in growing phototrophic cultures. Such approaches are of increasing importance for a reliable and in-real-time process monitoring and rapid adaptation of process conditions. In this study, spectroscopic probes supported by ultrasound particle traps were used as a viable technology for in-line, nondestructive, and real-time process analytics in photobioreactors. In details, spectroscopic attenuated total reflection Fourier-transformed mid-infrared (ATR-FTIR) spectra for P(3HB) and glycogen, two characteristic storage compounds in the selected model cyanobacterium *Synechocystis* sp. PCC 6714, were enhanced by ultrasonic particle manipulation, and used for *in situ* quantification of these compounds throughout the process [17].

Conversion of volatile fatty acids (VFAs), obtained by anaerobic transformation of organic residues, by mixed microbial cultures (MMCs) to PHA is another emerging tool to efficiently produce PHA from various waste streams [18]. Werker and colleagues addressed a well-known shortcoming of this approach: Feed streams of VFAs are typically highly diluted, thus preventing sufficient PHA productivity. To overcome this problem, authors established a novel flow-through bioprocess, which allows a trade-off between a maximum substrate inflow and a maximum substrate-to-PHA conversion (minimum outflow of not utilized substrate). To reach this goal, dynamics of upshift and downshift respiration kinetics were evaluated for MMCs on laboratory and pilot scale. Despite a constant influent substrate flow, biomass recycling into a mixing zone, which was engineered to contain higher substrate concentration, allowed for running the process at optimum PHA productivity, without compromising substrate utilization efficiency [19].

Kacanski and associates also addressed the fact that the typical high dilution of feed streams like VFAs solutions has a negative impact on productivity in fed-batch cultivation setups; the high feed stream volumes to be added into the bioreactor would excessively dilute the cultivation broth, and soon exceed the maximum allowable working volume in the bioreactor [20]. An option to solve this issue is to resort to cell retention systems, as shown before for PHA production on diverse highly diluted substrates in other studies [21,22,23,24]. In this *Bioengineering* Special Issue, Kacanski et al. presented a cell recycle fed-batch system for P(3HB) production by the versatile Gram-positive PHA producer *Bacillus megaterium* uyuni S29 from the VFA acetic acid. In this setup, a crossflow microfiltration membrane module was used to recycle active cell biomass (retentate fraction) into the bioreactor, while separating the permeate fraction (substrate-poor supernatant) from the system. An indirect pH-stat feeding regime, coupled to depletion of the substrate acetic acid, was successfully used for feed control in order to keep the substrate (acetate) concentration in an optimum range. A final biomass concentration of 19 g/L, harboring about 70 wt.% P(3HB), was obtained after 72 h of operation using this setup. The authors suggest follow-up studies to optimize the overall productivity by an enhanced nitrogen source feeding regime in order to further boost the concentration of biocatalytically active *Bacillus* cells for subsequent accumulation of PHA as a secondary, intracellular storage metabolite [20].

In addition to well-described *scl*-PHA, which consist of monomers made up by three to five carbon atoms, another emerging group of PHA biopolyesters was comprehensively reviewed for this Special Issue by Reddy and colleagues: the family of medium-chain-length PHAs (*mcl*-PHA), composed of monomers with six to 14 carbon atoms. Compared to *scl*-PHA, these biopolyesters are characterized by lower degree of crystallinity, glass transition temperature, and melting temperature. Different to *scl*-PHA production by strains like *C. necator*, which resorts to Class I PHA synthases, *mcl*-PHA biosynthesis requires special PHA synthase enzymes from Class II, and, in contrast to most representatives of *scl*-PHA, which have the properties of thermoplastics, *mcl*-PHA typically are elastomeric, latex-like, often sticky materials. Monomers building up *mcl*-PHA can be saturated or unsaturated; the latter, in turn, allows for post-synthetic modification by chemical or enzymatic reactions. The presented review article focuses on different carbon sources and at different operation modes for *mcl*-PHA production, on recent developments of methods for recovery of *mcl*-PHA from biomass, on their particular material properties, challenges during processing, applications in different sectors, and on their realized production on (semi)industrial scale in different global regions [25].

A completely new biopolyester type, structurally strongly related with PHA, was introduced years ago by the group of Alexander Steinbüchel in Münster: so-called poly(thioesters) (PTEs), which are produced by the enzymatic machinery (Class I PHA synthases) of established PHA-accumulating microbes when supplied with chemosynthetically produced mercaptoalkanoic acids [26]. It was demonstrated that, in contrast to PHA´s oxoester bonds, thioester bonds in PTEs are highly recalcitrant towards microbial degradation; only hybrid-type poly(oxo-thioesters) like poly(3-hydroxybutyrate-*co*-3-mercaptopropionate) (P(3HB-*co*-3MP)) or poly(3-hydroxybutyrate-*co*-3-mercaptobutyrate), but not PTE homopolyesters like poly(3MP) or poly(3MB) undergo biodegradation by PHA depolymerases, e.g., by depolymerase enzymes from *Schlegelella thermodepolymerans* [27]. In this Special Issue, Ceneviva and colleagues present the homopolythioester poly(3-mercapto-2-methylpropionate) (P(3M2MP)), a new α-methylated PTE with high rubber-like elasticity (elongation at break of 2600%), and its copolyester with 3HB (P(3HB-*co*-3M2MP)). The authors investigated biosynthesis of these new materials from 3-mercapto-2-methylpropionic acid as a structurally related precursor by engineered *Escherichia coli* LSBJ, and provided in-depth characterization of the material properties. It was shown that higher 3M2MP fraction in P(3HB-*co*-3M2MP) copolyesters caused lower molecular weight, and the materials became less crystalline, softer, more flexible, and revealed lower glass transition temperature and higher elongation at break, in addition to higher light transparency. Remarkably, it was shown that the rubber-like properties of this new PTE differ from those of previously described, not branched PTEs, evidencing that material properties can be further fine-tuned by introducing α-methylated thioester monomers. For follow-up studies, the authors plan to assess an eventual biodegradability of these materials [28].

After the biotechnological conversion of PHA-rich biomass, recovery of PHA from biomass by efficient, inexpensive, and sustainable approaches constitutes a key aspect of the entire PHA production process. In principle, methods for extraction of PHA from biomass, for digestion of the non-PHA biomass, or combinations thereof are described [29]. A novel PHA isolation approach based on the use of supercritical water (SBW) was developed by Meneses and colleagues. These authors used SBW at different temperatures to extract PHA from biomass of a mixed microbial culture cultivated on fruit waste in pilot scale setups. A temperature of 150 °C was feasible for readily decomposing the non-PHA biomass without excessively damaging PHA´s macromolecular structure. A polymer purity of 70% was obtained using SBW as sole PHA recovery agent; it was possible to increase purity to 80% by adding minor quantities of sodium hypochlorite solution for better solubilization of non-PHA biomass. Recovered biopolymer samples displayed some reduction in molecular mass and higher polydispersity than parallel samples extracted by the well-established, yet health-hazardous chloroform method. However, authors suggest that PHA obtained by the novel SBW-based recovery process might be of sufficient quality for those applications where excellent mechanical properties are not needed, such as use as additives, softeners or as low-molecular mass oligomers to be used for the preparation of graft or block copolymers [30].

The restricted processability of the homopolyester P(3HB), the best described and most frequently occurring natural PHA biopolyester, originates from its high crystallinity, brittleness, and low difference between the temperature of melting and onset of thermal degradation (too narrow “window of processability”). Addition of plasticizers is known a remedy to improve the mechanical properties of P(3HB) [31]. Such novel additives with antioxidative properties were developed and tested by Longé et al.; these authors esterified ferulic acid with butanediol, pentanediol, or glycerol, and used obtained esters for preparation of P(3HB)-based biocomposites by extrusion. Three hours after extrusion of pristine P(3HB) and the composites, respectively, it was shown that addition of ferulic acid esterified with butanediol to P(3HB) resulted in a strongly enhanced elongation at break from 11% (P(3HB)) to 270% for the composite consisting of 30% P(3HB) and 70% ester, reduced stress at break and Young´s modulus, and a ten-fold increase of the window of processability. Interestingly, this material improvement was not stable over time, but could be recovered by simple thermal treatment of the composite at a temperature below P(3HB)´s melting point, but just above the melting point of the butanediol ester of ferulic acid. Moreover, the high thermal stability of the additive further resulted in an increase in the fire retardancy property of the P(3HB)/ferulic ester composite material, while ferulic acid´s phenolic structure induced antioxidative properties as shown by radical scavenging tests. The authors suggest the need to study the suitability of prepared P(3HB)/additive composites, which are less hydrophobic than native P(3HB), for different packaging purposes [32].

Post-synthetic modification of microbial PHA to generate novel, functional materials becomes of increasing significance for improvement of performance of these bioproducts [33]. Brelle and colleagues prepared a novel class of *mcl*-PHA-based gels by using ionic interactions. First, highly hydrophilic sulfonated *mcl*-PHA derivatives were prepared from *mcl*-PHA samples with pending unsaturated side chains (poly(3-hydroxyoctanoate-*co*-33%-3-hydroxyundecenoate)); this was accomplished by thiol-ene reaction in presence of sodium-3-mercapto-1-ethanesulfonate. Such sulfonated *mcl*-PHA derivatives were crosslinked by bivalent inorganic cations or by ammonium derivatives of gallic acid or tannic acid. The nature of the cations highly determined the formation of the crosslinked networks. Using Ca^2+^ as bivalent cation, a network of low viscoelasticity was formed. With gallic acid derivatives, no network formation was possible, while the mechanical properties strongly increased in the presence of tannic acid derivates. Due to the presence of tannic acid, gels prepared this way displayed high antioxidant activity and remained stable for several months. The authors suggest these novel *mcl*-PHA-based networks for future use as active soft biomaterials in biomedical applications [34].

The final contribution to this Special Issue reviews current activities aiming at commercialization of PHA biopolyesters. Indeed, several companies have already started to produce and use PHA for a variety of applications. Currently, at least 25 companies and start-ups and more than 30 brand owners are declaring interest in PHA production and application. However, the combined product portfolio of currently active PHA manufacturing companies is still limited to five major types of PHA, namely the homopolyesters P(3HB) and poly(4-hydroxybutyrate) (P(4HB)), and the copolyesters poly(3-hydroxybutyrate-*co*-3-hydroxyvalerate) (P(3HB-*co*-3HV)), poly(3-hydroxybutyrate-*co*-4-hydroxybutyrate) (P(3HB-*co*-4HB)), and poly(3HB-*co*-3HHx), while only one company produces *mcl*-PHA of different composition on a reasonable scale as contract work. Optimistic capacity enlargements from currently estimated 1.5 million t over the next five years have been announced by different companies [35]. It will be fascinating to see the de facto developments on the PHA market during the next few years, in particular whether these enthusiastic industrial announcements can keep up with the actual increases in production and capacity!

## 3. Conclusions

The contributions to this Special Issue take readers on a journey into topical research activities in the realm of PHA biopolyesters. Aspects as manifold as microbiology, strain isolation, genetic engineering, process design and feeding regimes, in situ PHA quantification, inexpensive feedstocks, hetero- and autotrophic cultivation, novel downstream process strategies, *post synthesis* PHA modification, and preparation of compatible composites and blends are covered. As guest editor, I am optimistic that this third PHA-related *Bioengineering* Special Issue at hand will again spark inspiration and ideas for further research and development activities in the field of these fascinating biomaterials.
